# Class B1 GPCRs: insights into multireceptor pharmacology for the treatment of metabolic disease 

**DOI:** 10.1152/ajpendo.00371.2023

**Published:** 2024-07-10

**Authors:** Panjamaporn Sangwung, Joseph D. Ho, Tessa Siddall, Jerry Lin, Alejandra Tomas, Ben Jones, Kyle W. Sloop

**Affiliations:** ^1^Molecular Pharmacology, Lilly Research Laboratories, Eli Lilly and Company, Indianapolis, Indiana, United States; ^2^Department of Structural Biology, Lilly Biotechnology Center, San Diego, California, United States; ^3^Section of Cell Biology and Functional Genomics, Division of Diabetes, Endocrinology and Metabolism, Department of Metabolism, Digestion and Reproduction, Imperial College London, London, United Kingdom; ^4^Section of Investigative Medicine, Division of Diabetes, Endocrinology and Metabolism, Department of Metabolism, Digestion and Reproduction, Imperial College London, London, United Kingdom; ^5^Diabetes, Obesity and Complications, Lilly Research Laboratories, Eli Lilly and Company, Indianapolis, Indiana, United States

**Keywords:** glucagon-like peptide-1 receptor, glucagon receptor, glucose-dependent insulinotropic polypeptide receptor, obesity, type 2 diabetes

## Abstract

The secretin-like, class B1 subfamily of seven transmembrane-spanning G protein-coupled receptors (GPCRs) consists of 15 members that coordinate important physiological processes. These receptors bind peptide ligands and use a distinct mechanism of activation that is driven by evolutionarily conserved structural features. For the class B1 receptors, the C-terminus of the cognate ligand is initially recognized by the receptor via an N-terminal extracellular domain that forms a hydrophobic ligand-binding groove. This binding enables the N-terminus of the ligand to engage deep into a large volume, open transmembrane pocket of the receptor. Importantly, the phylogenetic basis of this ligand-receptor activation mechanism has provided opportunities to engineer analogs of several class B1 ligands for therapeutic use. Among the most accepted of these are drugs targeting the glucagon-like peptide-1 (GLP-1) receptor for the treatment of type 2 diabetes and obesity. Recently, multifunctional agonists possessing activity at the GLP-1 receptor and the glucose-dependent insulinotropic polypeptide (GIP) receptor, such as tirzepatide, and others that also contain glucagon receptor activity, have been developed. In this article, we review members of the class B1 GPCR family with focus on receptors for GLP-1, GIP, and glucagon, including their signal transduction and receptor trafficking characteristics. The metabolic importance of these receptors is also highlighted, along with the benefit of polypharmacologic ligands. Furthermore, key structural features and comparative analyses of high-resolution cryogenic electron microscopy structures for these receptors in active-state complexes with either native ligands or multifunctional agonists are provided, supporting the pharmacological basis of such therapeutic agents.

## INTRODUCTION

The class B family of cell surface, seven transmembrane-spanning G protein-coupled receptors (GPCRs) comprises receptors that possess architecturally diverse N-terminal extracellular domains (ECDs). For mammals, these GPCRs segregate into either the B1 secretin-like receptor subfamily of peptide hormone receptors or the B2 group of adhesion-type GPCRs, structurally related receptors that undergo auto-proteolysis of the ECD to enable receptor activation (1, 2). Although progress has been made over the past several years in investigating the unique mechanism of action of the adhesion GPCRs, along with the potential roles of these receptors in development, immune function, and certain types of cancer, at the moment, the B1 GPCRs have proven to be more therapeutically tractable as several drugs targeting these receptors are now approved by regulatory agencies (3, 4).

Among the most widely used therapies targeting class B1 receptors are agonists of the glucagon-like peptide-1 receptor (GLP-1R) for the treatment of type 2 diabetes (T2D) and obesity (5–8). The benefit of these medicines has recently inspired the development of new multifunctional ligands that activate additional members of the class B1 receptor family, such as the glucose-dependent insulinotropic polypeptide receptor (GIPR) and/or the glucagon receptor (GCGR), showing even greater efficacy for improving glycemic control and body weight management. The enhanced efficacy of either dual (GIP/GLP-1R or GLP-1/GCGR) or triple (GLP-1/GIP/GCGR) agonists versus mono-agonists of the GLP-1R is potentially due to the complementary nature of activating these additional receptor systems in key metabolic organs. In this article, we review the phylogenetic relationships of the GLP-1R, GIPR, and GCGR and highlight how the combined pharmacological activation of these GPCRs, and their respective signal transduction pathways, provides potential therapeutic benefits. Furthermore, recent high-resolution cryogenic electron microscopy (cryo-EM) studies revealing key ligand-receptor interactions are noted for the activity of the native ligands and the pharmacology of multifunctional agonists, including the GIP and GLP-1 receptor agonist, tirzepatide.

## CLASS B1 GPCRs

**Figure 1.**
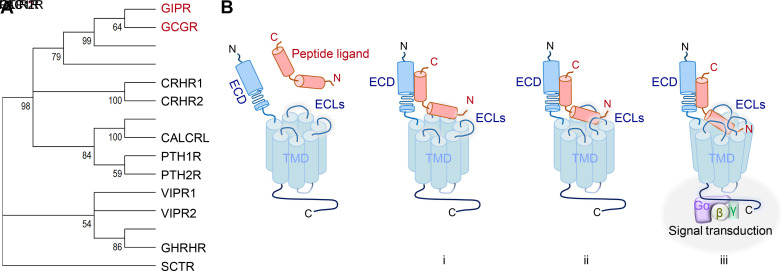
A phylogenetic tree of human class B1 G protein-coupled receptors (GPCRs) and a common activation (two-step binding) model. *A:* the phylogenetic tree of human class B1 GPCRs generated by the Maximum Likelihood method and Kimura 2 parameter model using MEGA 11 is depicted (9, 10). The numbers below the branches indicate the percentage of replicate trees in which the associated taxa is clustered together in the bootstrap test 1,000 replicates (11). Bootstrap values of 50% or lower are collapsed. Sequences used for the construction of phylogenetic tree were downloaded from NCBI/GenBank. *B i*: activation of the class B1 receptors is initiated by binding of the C-terminal region of the peptide ligand to the N-terminal extracellular domain (ECD) of the receptor, forming an affinity trap for the ligand. *ii*: this initial low affinity interaction brings the N-terminal region of the ligand to be positioned within close proximity of the extracellular loops (ECLs) and the 7 transmembrane domain (TMD) of the receptor. *iii*: the high-affinity engagement of the ligand with the ECD, ECLs, and TMD induces conformational changes of the receptor, thereby enabling G-protein coupling at the cytoplasmic side and inducing signal transduction.

The secretin-like, class B1 subfamily of GPCRs coordinate important physiological processes, including growth, the stress response, and glucose homeostasis. Members of this class of GPCRs were identified and characterized during the molecular biology revolution era of the 1990s. The class comprises 15 members (Fig. 1*A*) that contain structurally related features that are used in a common activation mechanism (Fig. 1*B*). These receptors consist of two distinct domains: an N-terminal ECD, followed by a seven transmembrane-spanning domain (TMD) (12, 13). The ECD is a globular trilayer structure of α-β-βα folds that are stabilized by three pairs of cysteine disulfide bonds (14). During engagement with the peptide ligand, the ECD of the receptor binds to the C-terminal portion of the ligand and serves as an “affinity trap” for the ligand, promoting the N-terminus of the ligand to bind deeply into a large volume, open pocket formed by the TMD, consequently eliciting receptor activation (Fig. 1*B*) (12, 13). Characterization of the native ligands for these receptors has led to the development of synthetically produced versions of some for medical diagnostic purposes and engineering of others for therapeutic use.

As the name recognizes, the “founding” member of the secretin-like, class B1 GPCRs is the secretin receptor (SCTR), first cloned in 1991 (15). The SCTR is expressed in the exocrine pancreas and other cell types of the gastrointestinal tract; binding of the hormone secretin promotes release of bile, bicarbonate, and pepsin to aid in digestion (16), and synthetic secretin is used in diagnosing pancreatic dysfunction (17, 18). Structurally similar members of the B1 family were then identified (Fig. 1*A*), revealing that these receptors regulate a diversity of physiological processes. For example, the identification of the corticotrophin-releasing hormone 1 (CRHR1) and 2 (CRHR2) receptors (19, 20) has helped characterize the regulation of the stress response through the hypothalamic-pituitary axis (CRHR1 activation by CRH1 stimulates secretion of adreno-corticotropic hormone) (21), in addition to various aspects of the cardiovascular and renal systems. A similar class B1 GPCR expressed in the pituitary is the growth hormone-releasing hormone receptor (GHRHR) (22), which helps regulate the synthesis and secretion of growth hormone. Notably, an analog of GHRH (sermorelin) is used for the treatment of growth hormone deficiency (23). In addition, discovery of the type 1 parathyroid hormone receptor (PTH1R) helped characterize calcium and phosphate homeostasis in the regulation of bone health (24, 25). The PTH1R is activated by parathyroid hormone–related protein (PTHrP) and by parathyroid hormone (PTH) of which an analog (teriparatide) is approved for the treatment of osteoporosis (25, 26). A closely related receptor, the type 2 parathyroid hormone receptor (PTH2R), is less-studied but has been implicated in nociception (27), various neuroendocrine activity (28), and calcium homeostasis (29). Another related receptor of therapeutic interest is the glucagon-like peptide 2 receptor (GLP-2R) (30). The GLP-2R is found in several locations, including the intestinal myenteric plexus, the brain, gastrointestinal tract (31), pancreatic α-cells (32), and hepatic stellate cells (33). It is a therapeutic target for the treatment of short bowel syndrome (SBS), and the GLP-2 analog, teduglutide, is on the market for the management of SBS (34).

One of the most intricate receptor signaling mechanisms in the GPCR field was revealed in studies of members of the class B1 family, the calcitonin receptor (CTR) and the calcitonin receptor-like receptor (CALCRL), which recognize the calcitonin/calcitonin gene‐related peptide (CGRP) family of peptides. The function of these receptors is altered by heterodimerizing with receptor activity-modifying proteins (RAMPs), forming six different receptor complexes. These include the CGRP (CALCRL/RAMP1), the amylin 1 (CTR/RAMP1), the amylin 2 (CTR/RAMP2), the amylin 3 (CTR/RAMP3), the adrenomedullin 1, and the adrenomedullin 2 (CALCRL/RAMP2 or RAMP3) receptors. Further details of these receptor complexes are comprehensively reviewed elsewhere (35, 36). Of note, both small molecule and monoclonal antibody antagonists targeting the CGRP receptor for migraine treatment have recently been developed (37–40).

Three closely related receptors, the pituitary adenylate cyclase-activating polypeptide 1 receptor (PAC1R) (41), the vasoactive intestinal polypeptide receptor 1 (VIPR1) (42), and the vasoactive intestinal polypeptide receptor 2 (VIPR2) (43), were also identified during this time period. PAC1R, VIPR1, and VIPR2 have high amino acid homology and mediate the biological responses of pituitary adenylate cyclase-activating peptide (PACAP) and vasoactive intestinal peptide (VIP), of which their amino acid sequences are ∼70% identical (44). These receptors are implicated in helping regulate numerous physiological responses, including inflammation, insulin secretion, and certain neurological functions (44, 45). However, as of now, there are no therapeutic analog of these ligands developed as treatments for disease conditions (3, 46).

While characterizing the aforementioned class B1 GPCRs has been important in enabling our understanding of their respective hormone systems, from a therapeutic standpoint, the GLP-1R (47), GIPR (48), and GCGR (49), three closely related class B1 family members, are proving to be effective drug targets, especially since single-peptide ligands that activate one or more of these receptors can be developed. Physiologically, the GLP-1R and GIPR are activated by the incretins, GLP-1 and GIP, which are secreted from entero-endocrine cells of the small intestines and act on their respective receptors to enhance insulin secretion in a glucose-dependent manner. The GCGR is predominantly expressed in the liver by hepatocytes (49), where it mediates the actions of glucagon to promote glucose output, as well as GCGR agonist-driven energy expenditure (50, 51). Beyond the liver, the GCGR is also expressed in the kidney and in pancreatic islets (52). Comparing the three receptors, ∼40% and 44% of residues comprising the GIPR are identical to those of the GLP-1R and the GCGR, respectively (48, 53). Similarly, the amino acid identity between the GCGR and the GLP-1R is ∼42%. In addition to pancreatic β-cells, the GLP-1R is expressed in areas of the central nervous system (CNS) and kidney, and in various cell types of the gastrointestinal tract (54). Likewise, the GIPR is also found in other cells, including pancreatic islet α-cells, the CNS, and cell types of the gastrointestinal tract (48). Therapeutically, the first drugs developed targeting these receptors were mono-agonists of the GLP-1R, including liraglutide (55), dulaglutide (6), and semaglutide (8).

Recently, drugs targeting two or all three of these receptors have emerged, with tirzepatide (a GIP and GLP-1 receptor agonist) being the first to gain regulatory approval for the treatment of T2D (56–58). Other promising multifunctional agonists that are currently in late stages of development include GLP-1/GCGR agonists such as efinopegdutide (59, 60), mazdutide (61, 62), pemvidutide (63, 64), and survodutide (65, 66), along with the triple agonists efocipegtrutide (67) and retatrutide (68). For these class B1 GPCRs, incorporating GIPR and/or GCGR pharmacology with GLP-1R activity enables the stimulation of additional cell signaling pathways, thereby inducing complementary mechanisms to treat metabolic disease. At the moment, efinopegdutide is being investigated in a phase 2 b study for the treatment of nonalcoholic steatohepatitis (NASH) (NCT05877547). Mazdutide is in phase 3 trials for the treatment of obesity (NCT05607680) and T2D (NCT05628311 and NCT05606913). For pemvidutide, a phase 2 study for obesity (NCT05295875) has been completed, whereas one in NASH (NCT05989711) is ongoing. Survodutide has completed phase 2 studies for both obesity (NCT04667377) and T2D (NCT04153929) (phase 3: NCT06066515, NCT06066528), whereas a trial in NASH (NCT04771273) is ongoing. Finally, efocipegtrutide is in development to treat NASH (NCT04505436), and retatrutide is being investigated in phase 3 programs for the treatment of obesity (NCT05929066) and also T2D (NCT06354660).

## SIGNAL TRANSDUCTION AND TRAFFICKING OF THE GLP-1, GIP, AND GLUCAGON GPCRs

As indicated earlier, for class B1 GPCRs, receptor activation occurs via a two-step mechanism where the peptide ligand is initially recognized by an N-terminal ECD of the receptor (13). Ligand binding promotes conformational changes in the receptor that trigger the engagement of downstream effectors including heterotrimeric G proteins, which are comprised of a Gα subunit and a Gβ/γ dimer that is reversibly bound to Gα. Gα subunits can be further classified into four main subtypes: Gαs, Gαi/o, Gαq/11, and Gα12/13, based on their interaction with downstream effectors (69). Heterotrimeric G proteins play a critical role in transmitting extracellular signals toward intracellular responses, with specifically induced cellular responses depending upon the activated G protein subtype and the downstream effectors involved. Although Gαs and Gαq/11 are classically known to stimulate second messenger production (70) [such as cyclic adenosine monophosphate (cAMP) following adenylyl cyclase (AC) activation for Gαs and diacylglycerol (DAG) and inositol trisphosphate (IP3) following phospholipase Cβ (PLCβ) activation for Gαq/11], Gαi is known to inhibit AC activity and therefore reduces cAMP production (71). Importantly, spatiotemporal modulation of distinct downstream signaling pathways enables GPCRs to exert precise pleiotropic control over a diverse range of cellular outputs (72).

**Figure 2.**
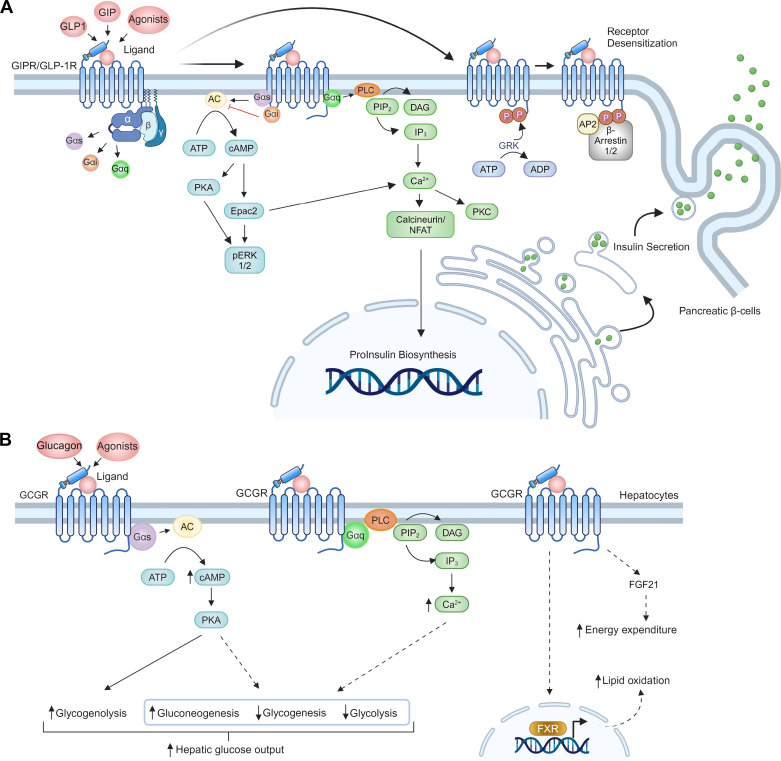
Signal transduction of the glucagon-like peptide-1 (GLP-1), glucose-dependent insulinotropic polypeptide (GIP,) and glucagon G protein-coupled receptors (GPCRs). *A*: glucagon-like peptide-1 receptor (GLP-1R) and glucose-dependent insulinotropic polypeptide receptor (GIPR) signaling and downstream pathway activation in pancreatic β-cells. Incretin receptor stimulation can lead to Gαs and Gαq coupling, leading to upregulation of cyclic adenosine monophosphate (cAMP) and intracellular Ca^2+^, which results in downstream insulin production and secretion. *B*: activation of the glucagon receptor (GCGR) in hepatocytes enhances hepatic glucose output, lipid oxidation, and energy expenditure. Figure 2 was created with BioRender.com.

Classically, the GLP-1R has been reported to preferentially couple to Gαs, followed by Gαq, with minimal coupling to Gαi in response to GLP-1 stimulation (73). Upon binding of GLP-1, the GLP-1R couples to the heterotrimeric G protein complex and induces nucleotide exchange of GDP to GTP on the Gα subunit. GTP binding to Gαs causes dissociation from Gβ/γ, thereby enabling availability to downstream effectors. GTP-bound Gαs stimulates AC to catalyze the conversion of adenosine triphosphate (ATP) to cAMP, leading to the activation of downstream effectors such as protein kinase A (PKA) and exchange protein activated by cyclic AMP 2 (Epac2). The signal transduction terminates upon the hydrolysis of GTP to GDP on the Gαs subunit, which restores the heterotrimeric G protein complex, making it available for another G-protein coupling event with the receptor (74). In pancreatic β-cells, these interactions initiate downstream signaling cascades that ultimately elicit glucose-dependent insulin secretion (Fig. 2*A*). The importance of Gαq-dependent signaling, in contrast, was recently found to be amplified under physiological GLP-1 levels (∼30 pmol/L) versus nanomolar concentration of GLP-1, which would primarily induce Gαs coupling (75). Such a hypothesis is supported by studies reporting that picomolar concentrations of GLP-1 can stimulate insulin secretion without a significant accumulation of cAMP, an effect diminished by calcium channel blockers such as verapamil and dantrolene (76, 77). These findings imply a new mechanism of physiological GLP-1R action, independent of the cAMP-PKA pathway, with the latter pathway presumably more relevant under pharmacological conditions.

Until recently, little was known about the signaling characteristics of the GIPR. A study by Manchanda et al. (73) elucidated the distinct molecular mechanisms that dictate divergent insulinotropic effects of stimulation of the GLP-1R and GIPR in a rat pancreatic β-cell line. This study showed that coupling responses to the GIPR were markedly reduced compared with those of the GLP-1R for all signaling mediators probed: β-arrestin 2, Gαs, Gαi, and Gαq. Despite marked reductions in Gα subunit coupling, GIPR stimulation enhanced downstream signaling outputs, indicating a higher degree of signal amplification for this receptor, results in-line with GIP being characterized as the predominant incretin (highlighted in the section therapeutic roles of glp-1, gip, and glucagon gpcrs in metabolic control). Marked differences between the signaling characteristics of class B1 GPCRs in pancreatic β-cells may hint at distinct coupling characteristics being present in other tissues where these receptors are also expressed. Although signaling and trafficking signatures of class B1 GPCRs in other cell types, including islet α-and δ-cells and the CNS, are not yet elucidated, future research on specific receptor coupling profiles could help explain their divergent effects, for example, opposed α-cell glucagonotropic signatures of the GLP-1R versus the GIPR. Although studies have also characterized the insulinotropic effect of glucagon via the GCGR in β-cells (78), the majority of work on the GCGR has focused on hepatocytes where glucagon binding induces Gαs signaling (79), but GCGR coupling to Gαq is also well-established (80) (Fig. 2*B*). Activation of the GCGR promotes hepatic glucose output, along with lipid oxidation and energy expenditure, although the full signaling pathways inducing the latter are less understood.

As with most ligand-activated receptors, class B1 GPCRs undergo endocytosis as a result of ligand binding and activation, a phenomenon that contributes to the downregulation of receptor signaling and control of receptor levels at the plasma membrane. Postendocytic receptor destinations include lysosomal degradation, which leads to termination of signaling, or recycling back to the cell surface, enabling resensitization and sustained signaling. In addition to the differences in coupling propensities discussed earlier, marked differences in receptor trafficking have also been observed between the GLP-1R and the GIPR, with the former associated with a fast internalizing, slow recycling profile, whereas the latter shows reverse trafficking characteristics when activated by their corresponding native ligands (73). Although similar trafficking profiles have not been determined for the GCGR in primary cells, studies in HEK293T and human hepatoma (Huh7) cells have shown that this receptor is associated with a slow internalization and fast recycling profile in response to glucagon stimulation, with a report demonstrating intracellular retention of GCGR being triggered by RAMP2 coexpression (81).

The endocytic pathway consists of a network of intracellular vesicular compartments, with each interconnected subsequent compartment displaying a higher level of maturation (82). The high level of compartmentalization of the endocytic pathway, along with the discovery that GPCRs are capable of sustaining intracellular signaling from different postendocytic destinations following their internalization (83), points toward selectivity in downstream responses being achieved at least in part by localized signaling, either by controlling access to specific signaling nodes present in these intracellular locations or through the interplay between distinct intracellular organelles including, for example, endosomes, the Golgi apparatus, the endoplasmic reticulum, and/or mitochondria (84, 85). This has led to the development of the concept of nanodomain-organized signal compartmentalization (86), whereby a discrete area of a few nanometers in size, harboring its own specific lipid and protein composition (“nanodomain”), determines GPCR behavior by organizing receptors and effectors into signaling “hubs” (87–89). For the GLP-1R, a high degree of signal compartmentalization has been demonstrated in heterologous cell systems involving plasma membrane nanodomains that contain the machinery required for the activation of the cAMP-dependent PKA pathway, thus enabling spatiotemporal control of GLP-1R activity (72). This is supported by studies demonstrating active GLP-1R segregation to cholesterol-rich plasma membrane nanodomains in pancreatic β-cells (90). Whether signaling from the GIPR or the GCGR is equally structured in signaling nanodomains, along with the consequences of multifunctional agonism at these receptors, remains to be investigated.

## THERAPEUTIC ROLES OF THE GLP-1, GIP, AND GLUCAGON GPCRs IN METABOLIC CONTROL

Sustained pharmacological activation of each of the GLP-1R, GIPR and GCGR results in wide-ranging metabolic effects. Initially developed as treatments for T2D, GLP-1R mono-agonists exert effective blood glucose-lowering via several mechanisms. Examples of these drugs include Victoza (liraglutide; acylated with palmitic acid for once-daily dosing; FDA approved in 2010) (55), Trulicity (dulaglutide; fused to the Fc component of immunoglobulin G4 heavy chain for once-weekly dosing; FDA approved in 2014) (6), and Ozempic (semaglutide; acylated with C18 diacid for once-weekly dosing; FDA approved in 2017) (8). Within the pancreatic islet, direct β-cell stimulation by GLP-1R agonists promotes insulin secretion (91), whereas glucagon release is inhibited. The latter might require GLP-1R agonists to act initially on δ-cells, in turn suppressing α-cell activity via paracrine somatostatin (92). It is estimated that the insulinotropic and glucagonostatic effects of GLP-1 contribute equally to blood glucose regulation (93). In short-term studies, the glucose-lowering effects of GLP-1 and therapeutic GLP-1R agonists depend heavily on delayed nutrient absorption via slowing of gastric emptying (94), but these effects are subject to tachyphylaxis and diminish over time (95, 96). A major driver of pharmacological GLP-1R agonist antihyperglycemic efficacy is insulin sensitization via weight loss (97). GLP-1R agonist-mediated weight loss is associated with reduced energy intake, mediated primarily via anorectic CNS GLP-1Rs in key neuronal populations that are located in regions accessible to ligands in the peripheral circulation (98, 99). GLP-1R-expressing neurons in the hypothalamic arcuate nucleus and the dorsovagal complex in the brainstem are particularly implicated (100, 101). However, brain GLP-1R activation can also lead to nausea and vomiting. Although these effects tend to decline with time and can be partly avoided by dose escalation schedules, they impose a maximum dose limit, which likely prevents achieving the optimum therapeutic benefit of these agents.

In contrast, the GIPR has not always attracted attention as a therapeutic target. GIP is the dominant incretin in healthy individuals (102), but its effect has been reported to be reduced in the context of diabetes (103), originally limiting its potential appeal as a therapeutic antihyperglycemic agent. Unlike GLP-1, GIP enhances glucagon secretion (104). Notably, administration of native GIP fails to suppress appetite in humans in short-term studies (105, 106), and some lines of genetic evidence suggest that GIPR inactivation, rather than activation, exerts body weight-lowering effects (107, 108). There is now a developing view that GIPR mono-agonists can suppress appetite and reduce body weight (109–112), although most of these results are from preclinical studies. However, a recent study has demonstrated that a long-acting GIPR mono-agonist can produce weight loss, although modest, in humans (113). While multifunctional analogs, including tirzepatide and retatrutide, incorporate GIPR agonism and show impressive weight loss, so does the GLP-1R agonist/GIPR antagonist AMG-133; however, the multiplicity of target receptors (GLP-1R, GIPR, and GCGR) for these ligands means that elucidating the contribution of GIPR action to their therapeutic effects in humans is challenging. It is suggested that the paradoxical ability of both GIPR agonists and antagonists to reduce body weight can be reconciled by sustained GIPR agonism leading to target downregulation, thereby functionally mimicking GIPR antagonism (114), although this remains to be tested in the CNS. Chronic blockade of the GIPR could also be compensated by an increase in the responsiveness of GLP-1R signaling, thereby enabling a greater GLP-1R agonist effect. Further possible benefits of GIPR agonism include an anti-emetic effect, which might allow higher dosing equivalents of GLP-1 by countering gastrointestinal effects (115) and promoting weight-independent improvement of insulin sensitivity (116). Some of the most compelling evidence supporting the benefit of GIP/GLP-1R agonism comes from a 40-wk, head-to-head study showing the superiority of tirzepatide over semaglutide for glucose and body weight reduction in patients with T2D (58). The outcome of improved glucose control by treatment with tirzepatide aligns with recent findings from studies of human islets showing that robust insulinotropic effects of tirzepatide can occur even when pharmacologically blocking either receptor (117), supporting the notion that activating both incretin receptors may be therapeutically advantageous. Tirzepatide (acylated with C20 diacid for once-weekly dosing; FDA approved in 2022 for the treatment of T2D) (56) is marketed as Mounjaro.

The GCGR as a target is somewhat different from GLP-1R and GIPR, as its purported benefits relate less to stimulation of insulin secretion and anorectic weight loss (although some studies report these effects) (118, 119) and more to promoting weight loss via increasing energy expenditure. The thermogenic effect of glucagon has long been recognized (120), but its potential therapeutic value was neglected for many years because of the perception that its blood glucose-elevating tendency (via hepatic glycogenolysis and gluconeogenesis) precluded application as a treatment for T2D. Early work using the endogenous GLP-1/GCGR agonist oxyntomodulin (OXM) demonstrated that a useful GCGR effect on energy expenditure can be achieved without raising blood glucose (121, 122). Similar effects are observed in obese mice treated with retatrutide, where reduced energy expenditure from the anorectic effect of GIP/GLP-1R agonism can be countered by the GCGR pharmacology of the triple agonist (68). The precise mechanism for GCGR-mediated thermogenesis is incompletely understood, but most likely arises through acceleration of ATP-dependent metabolic processes in the liver and the induction of energetic demand in other key metabolic organs (123, 124). The complexity of these effects is illustrated by the observation that, at least in mice, they are also dependent on FGF21 and FXR signaling (125, 126), but there are probably also FGF21-independent mechanisms, especially in primates. A possible role for direct stimulation of brown adipose tissue has been largely discounted (127, 128). A second important benefit of GCGR agonism is its ability to clear liver fat, raising hopes it could be used to treat NASH (129, 130). Indeed, a barrier to the historic development of GCGR antagonists as antihyperglycemic agents for diabetes was their tendency to promote hepatic steatosis (131). Importantly, antisteatotic effects of glucagon depend on the hepatic GCGR and may be achieved in the absence of weight loss (132). An additional consideration for glucagon is around its potential to accelerate amino acid-consuming ureagenesis in the liver, prompting hypoaminoacidemia (133). The issue is that this could contribute to the loss of lean body mass, a symptom of sustained hyperglucagonemia in the glucagonoma syndrome (134, 135). However, it has been shown that, in mice, amelioration of hepatic steatosis is possible at doses that do not affect protein catabolism. Importantly, readouts of ongoing clinical trials for molecules possessing GCGR activity will help inform on the desirable pharmacological balance of GCGR versus GLP-1R agonism (and GIPR in the cases of efocipegtrutide and retatrutide). Although head-to-head assays evaluating the relative potency ratios of these molecules have not been published, primary reports suggest that efinopegdutide (59), mazdutide (61, 62), pemvidutide (136), and survodutide (65) display generally balanced activity at both the GCGR and the GLP-1R for inducing cAMP signaling. For retatrutide, it is also fairly balanced at these receptors but displays relatively stronger potency at the GIPR (68).

## STRUCTURAL COMPARISON OF THE GLP-1, GIP, AND GLUCAGON GPCRs

**Figure 3.**
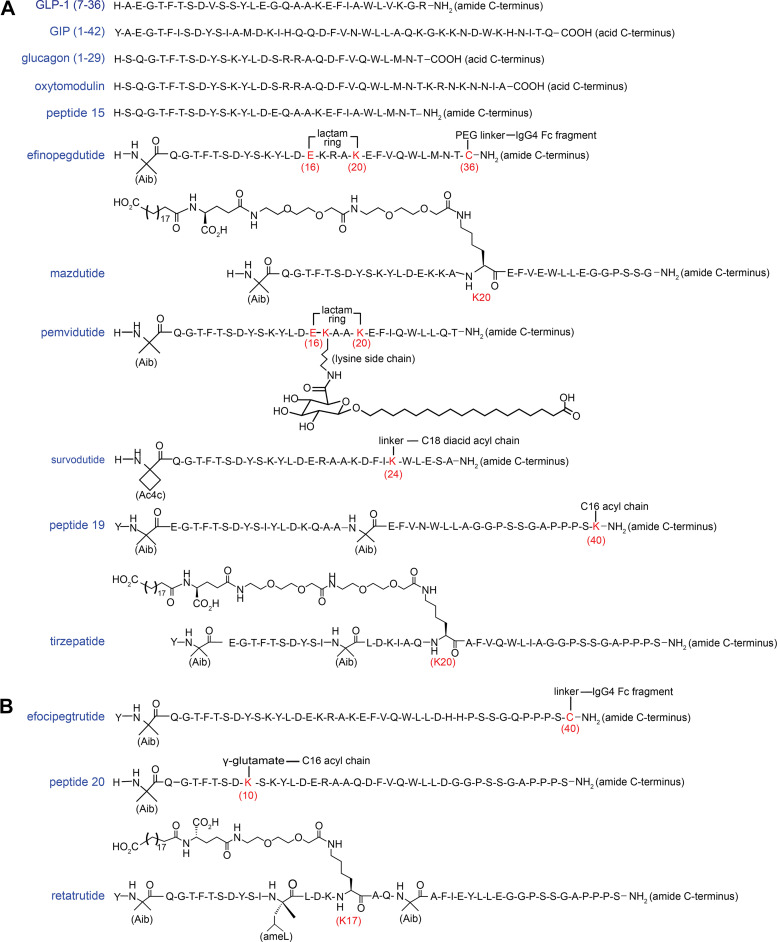
Peptide sequence of native peptide agonists, dual agonists (*A*), and triple agonists (*B*) that have been studied using cryogenic electron microscopy (cryo-EM) or are currently in late stages of clinical development. Unnatural amino acids are indicated as blue text: Aib, alpha-amino isobutyric acid; Ac4c, 1-amino-cyclobutanecarboxylic acid; ameL, alpha-methyl-L-leucine. Residues with covalent modifications are indicated as red text. Glucagon-like peptide-1 (GLP-1)/ glucagon receptor (GCGR) agonists: oxyntomodulin (OXM): MW 4422.9 Da (142); peptide 15 (pep-15): MW 3356.6 Da (143); efinopegdutide (MK-6024, HM12525A): side chains of E16 and K20 forming a macrocycle and an IgG4 Fc region with a polyethylene glycol linker attached at C36. (144); mazdutide (IBI362, LY3305677): C20 diacid acyl moiety with linker attached at K20, MW 4563.1 Da (145, 146); pemvidutide (ALT-801, SP-1373): side chains of E16 and K20 forming a macrocycle with a glucuronic acid/C18 diacid acyl moiety attached at K17, MW 3873.4 Da (63, 64); survodutide (BI 456906): C18 diacid acyl moiety with linker attached at K24, MW 4231.6 Da (65, 66). glucose-dependent insulinotropic polypeptide (GIP)/ glucagon-like peptide-1 receptor (GLP-1R) agonists: peptide 19 (pep-19): C16 monoacid acyl moiety attached at K40, no linker, MW 4473.1 Da (147); tirzepatide (TZP; LY3298176): C20 diacid acyl moiety with linker attached at K20, MW 4813.5 Da (56). GLP-1/GIP/GCGR agonists: efocipegtrutide (HM15211): an IgG4 Fc region with a polyethylene glycol linker attached at C40 (67). The sequence of efocipegtrutide is reported in WHO Drug Information, Vol. 35, No. 4, 2021, Proposed INN: List 126. peptide 20 (pep-20): C16 diacid acyl moiety with linker attached at K10), MW 4543.1 Da (148); retatrutide (LY3437943): C20 diacid acyl moiety with linker attached at K17, MW 4731.4 Da (68).

A major technological advance in investigating the specificity of ligand recognition and receptor activation for class B1 GPCRs has been the application of cryo-EM for solving near-atomic resolution (4 Å or better) structures of these receptors in the ligand-bound, active state conformation (137–141). As such, to help understand the pharmacological basis for activating the GLP-1R, GIPR, and GCGR, cryo-EM was used to obtain high-resolution structures of these receptors in complex with their native ligands or either dual or triple agonists (Fig. 3; Table 1). Comparing the cryo-EM structures of the GLP-1R, GIPR, and GCGR bound to their respective mono-agonists, the contact residues of the receptor that engage the ligand have a minimum of 40% sequence identity and 60% sequence similarity (Table 2). Furthermore, the three native ligands (GLP-1, GIP, and glucagon) share a minimum of 38% sequence identity and 62% sequence similarity (OXM shares the same N-terminal 29 residues of glucagon) (Fig. 3). In part, the sequence similarity among GLP-1, GIP, and glucagon propelled the field to research and develop dual and triple agonists for investigating the potential benefit of receptor polypharmacology. Overall, at the “atomic” level, when comparing mono-agonist-bound GLP-1R/GIPR/GCGR structures to some of the dual agonist- and triple agonist-bound structures, there is remarkable similarity in the TMDs of the receptors, but notable differences are found in the three extracellular loops (ECL1, 2, 3) and in the juxtaposition regions of the ECDs to the TMDs (further discussed below).

**Table 1.** GLP-1R, GIPR, and GCGR cryo-EM structures bound to their respective native ligands, dual agonists, and triple agonists

Peptide Agonist	Pharmacology	PDB (Resolution, Receptor)	Publication
GLP-1	GLP-1R endogenous agonist	6X18 (2.10 Å, GLP-1R) 5VAI (4.10 Å, GLP-1R)	(149, 150)
GIP	GIPR endogenous agonist	7RA3 (3.24 Å, GIPR) 7DTY (2.98 Å, GIPR)	(151, 152)
Glucagon	GCGR endogenous agonist	6LMK (3.70 Å, GCGR)	(153)
OXM	GLP-1/GCGR endogenous agonist	7LLY (3.30 Å, GLP-1R)	(154)
Pep-15	GLP-1/GCGR agonist	6WHC (3.40 Å, GCGR) 8JIQ (2.50 Å, GCGR) 8JIS (2.46 Å, GLP-1R)	(155, 156)
MED10382 (cotadutide)	GLP-1/GCGR agonist	8JIP (2.85 Å, GLP-1R) 8JIT (2.91 Å, GCGR)	(156)
SAR425899	GLP-1/GCGR agonist	8JIR (2.57 Å, GLP-1R) 8JIU (2.76 Å, GCGR)	(156)
Pep-19	GIP/GLP-1R agonist	7RTB (2.14 Å, GLP-1R)	(157)
Pep-20	GLP-1/GIP/GCGR agonist	7VBH (3.00 Å, GLP-1R) 7FIN (3.10 Å, GIPR) 7V35 (3.50 Å, GCGR)	(158)
TZP	GIP/GLP-1R agonist	7RGP (2.90 Å, GLP-1R) 7RBT (3.08 Å, GIPR) 7FIM (3.40 Å, GLP1R) 7FIY (3.40 Å, GIPR)	(151, 158)

GCGR, glucagon receptor; GIPR, glucose-dependent insulinotropic polypeptide receptor; GLP-1R, glucagon-like peptide-1 receptor; OXM, oxyntomodulin; pep-15, peptide 15; pep-19, peptide 19; pep-20, peptide 20; TZP, tirzepatide.

**Table 2.** Amino acid sequence identity/similarity comparisons of GLP-1, GIP, and glucagon and the peptide-binding pocket residues of their respective receptors

	GLP-1R + GLP-1 Complex (PDB: 6X18)	GIPR + GIP Complex (PDB: 7RA3)	GCGR + Glucagon Complex (PDB: 6LMK)
GLP-1R + GLP-1 complex (PDB: 6X18)		47%/60% (receptors) 38%/62% (peptides)	42%/60% (receptors) 48%/76% (peptides)
GIPR + GIP complex (PDB: 7RA3)	47%/60% (receptors) 38%/62% (peptides)		44%/69% (receptors) 48%/66% (peptides)
GCGR + glucagon complex (PDB: 6LMK)	42%/60% (receptors) 48%/76% (peptides)	44%/69% (receptors) 48%/66% (peptides)	

The amino acids of the peptide-binding pockets are selected to be within a distance of 4.5 Å from the native ligand. The calculated surface areas that are buried by the bound ligands are 1466 Å^2^, 1226 Å^2^, and 1416 Å^2^ for GLP-1R, GIPR, and GCGR, respectively. GCGR, glucagon receptor; GIP, glucose-dependent insulinotropic polypeptide; GIPR, glucose-dependent insulinotropic polypeptide receptor; GLP-1R, glucagon-like peptide-1 receptor; OXM, oxyntomodulin.

**Figure 4.**
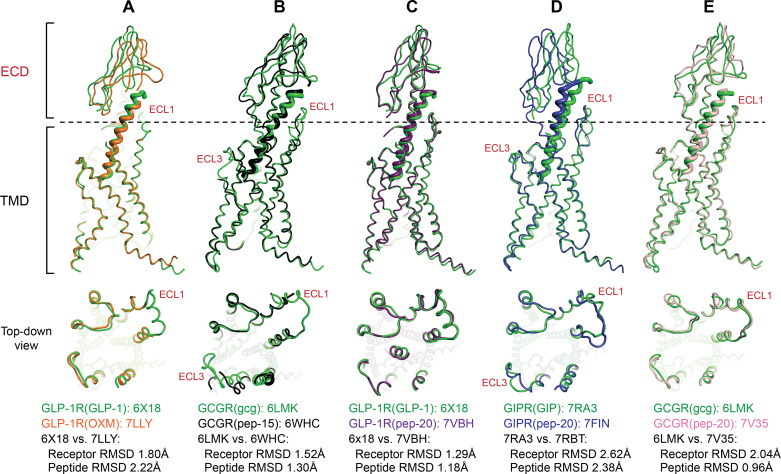
Structural comparison of the glucagon-like peptide-1 receptor (GLP-1R), glucose-dependent insulinotropic polypeptide receptor (GIPR), and glucagon receptor (GCGR) bound to their native ligands [glucagon-like peptide-1 (GLP-1), glucose-dependent insulinotropic polypeptide (GIP), glucagon], dual agonists [oxyntomodulin (OXM), pep-15], and triple agonist (pep-20). *A*: GLP-1R bound to GLP-1 vs. OXM. *B*: GCGR bound to glucagon vs. pep-15. *C*: GLP-1R bound to GLP-1 vs. pep-20. *D*: GIPR bound to GIP vs. pep-20. *E*: GCGR bound to glucagon vs. pep-20). The 4-letter Protein Data Bank (PDB) codes are indicated in the figure. Receptors are shown as thin lines; ligands are shown as pink ribbons. Major area of difference indicated in red text. Top-down view (from the extracellular side): extracellular domain (ECD) and ligand are excluded for clarity. Pairwise comparisons of the Cα root mean square deviation (RMSD) between receptors and ligands are indicated beneath. Cα RMSDs are calculated by aligning the Cα of the receptor, excluding the ligand.

Although cryo-EM studies for the GLP-1/GCGR agonists that are currently in clinical development (such as efinopegdutide, mazdutide, pemvidutide, or survodutide) have yet to be reported, high-resolution structures of OXM and peptide 15 (pep-15) bound to these receptors provide insights supporting this co-agonist mechanism (142,143, 159). In the GLP-1R, comparing the receptor conformations of OXM bound versus GLP-1 bound, ECL1 becomes disordered, which is accompanied by a large shift in the juxtaposition of the ECD in respect to the TMD (Fig. 4*A*) (154). In the GCGR, comparing the receptor conformation of pep-15 bound versus glucagon bound, disorder is observed in the ECL1 and ECL3 of the receptor when bound to pep-15 (Fig. 4*B*) (155). The authors of this report also note that the ECD is less resolved in the OXM-bound GLP-1R and pep-15-bound GCGR structures, suggesting increased conformational dynamics at the ECD due to weaker interaction with the C-terminal region of the ligand (154, 155). Recently, the structures of two other GLP-1/GCGR agonists, MED10382 (cotadutide) and SAR425899, bound to GLP-1R and GCGR have been reported (156); the authors indicate a notable difference in the ECL3 where MED10382 and SAR425899 induce a more inward movement in comparison with pep-15 (156). Clinical programs investigating both of these molecules have stopped, but the cryo-EM structures of these ligands in complex with the GLP-1R and the GCGR provide further evidence underpinning the complexity of discovering dual receptor agonists.

Peptide 20 (pep-20; closely related to NNC9204-1706/MAR423) is a triple agonist at the GLP-1R, GIPR, and GCGR. Although development of this molecule as a therapeutic agent was unsuccessful, cryo-EM studies of pep-20 in complex with the three receptors provide insights into the triple agonist mechanism. At the GIPR and GCGR, pep-20 elicits bias toward G-protein coupling while maintaining balanced signaling at the GLP-1R (158, 160). Comparing structures of the GLP-1R bound to pep-20 versus the GLP-1, ECL1 and ECL3 adopt the same conformation, and there is little change in the juxtaposition between the ECD and TMD (Fig. 4*C*). However, differences in the ECL1 and ECD are observed when pep-20 is bound to the GIPR versus GIP (Fig. 4*D*). In the GCGR bound to pep-20 versus native glucagon, there is only slight alteration in the ECL1 and ECD (Fig. 4*E*). Overall, analyzing the root mean square deviations (RMSDs) of pep-20-bound GLP-1R, GIPR, and GCGR structures to their respective native peptide-bound structures, pep-20 induces the largest change in the GIPR, followed by the GCGR and the GLP-1R (RMSDs are 1.29 Å for the GLP-1R, 2.62 Å for the GIPR, and 2.04 Å for the GCGR) (Fig. 4, *C*–*E*). Notably, the field awaits reporting of cryo-EM structures for the triple agonists efocipegtrutide and retatrutide.

**Figure 5.**
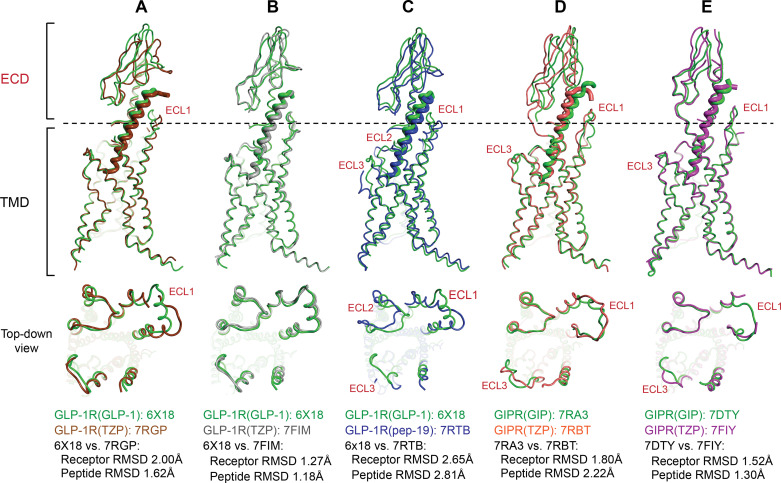
Structural comparison of the glucagon-like peptide-1 receptor (GLP-1R) and the glucose-dependent insulinotropic polypeptide receptor (GIPR) bound to their native ligands [glucagon-like peptide-1 (GLP-1), glucose-dependent insulinotropic polypeptide (GIP)] and dual agonists [tirzepatide (TZP), pep-19]. *A*: GLP-1R bound to GLP-1 vs. TZP. *B*: GLP-1R bound to GLP-1 vs. TZP (the alternate structure). *C*: GLP-1R bound to GLP-1 vs. pep-19. *D*: GIPR bound to GIP vs. TZP. *E*: GIPR bound to GIP vs. TZP. The 4-letter Protein Data Bank (PDB) codes are indicated in the figure. Receptors are shown as thin lines; ligands are shown as pink ribbons. Major area of difference indicated in red text. Top-down view (from the extracellular side): extracellular domain (ECD) and ligand are excluded for clarity. Pairwise comparisons of the Cα root mean square deviation (RMSD) between receptors and ligands are indicated beneath. Cα RMSDs are calculated by aligning the Cα of the receptor, excluding the ligand.

Tirzepatide and peptide 19 [pep-19; closely related to NNC0090-2746/MAR709 (161)] are GIP and GLP-1 receptor agonists (56, 147). In the protein data bank (PDB), two tirzepatide-bound GLP-1R and two GIP-bound GIPR cryo-EM structures have been deposited (151, 158), with the structures from the two contributing research groups exhibiting notable differences when overlaid. The differences in the reported cryo-EM structures are likely a result from extraneous variables in construct designs and cryo-EM data processing protocols (151, 158). To minimize contributions of the potential variables, the structures from the same research group are compared. When comparing the two tirzepatide-bound GLP-1R structures to the GLP-1-bound structure, tirzepatide induces changes in the ECL1 and ECD, but this is only observed when comparing 6X18 with 7RGP (Fig. 5, *A* and *B*) (151, 158). Considering receptor RMSDs when tirzepatide is bound versus native peptide, tirzepatide induces less changes to the GIPR than to the GLP-1R. GLP-1R RMSD bound to tirzepatide versus GLP-1 is 2.00 Å [comparing 6X18 with 7RGP in the study by Sun et al. (151)] and 1.27 Å [comparing 6X18 with 7FIM in the study by Zhao et al. (158)], respectively (Fig. 5, *A* and *B*). GIPR RMSD bound to tirzepatide versus GIP is 1.80 Å [comparing 7RA3 with 7RBT in the study by Sun et al. (151)] and 1.52 Å [comparing 7DTY with 7FIY in the study by Zhao et al. (152, 158)] (Fig. 5, *D* and *E*). At the peptide sequence level, the N-terminal Tyr 1 residue of tirzepatide appears to also induce unique pharmacology at the GLP-1R. Although tirzepatide acts as a G-protein-biased agonist at the GLP-1R, mutating the N-terminus of tirzepatide from Y1 to H1 significantly increases the activity of the peptide at the GLP-1R and restores some of the ability of GLP-1R to recruit β-arrestin (151); mechanistically, the structural studies indicate that Y1 in tirzepatide disrupts a stable interaction between TM5 and ECL3 of the GLP-1R, and the Y1H mutation likely restores this interaction, thereby enabling the receptor to more readily recruit β-arrestin. On the contrary, pep-19 is not G-protein biased at the GLP-1R (160). Comparing pep-19 versus GLP-1-bound GLP-1R structures, ECL1 adopts a different secondary structure, whereas both ECL2 and ECL3 open more outwardly when pep-19 is bound to the GLP-1R (Fig. 5*C*). To more extensively analyze the cryo-EM datasets in the GLP-1R structures bound to pep-19 and GLP-1, Johnson et al. (157) performed 3-D variability analysis [3DVA, an algorithm within cryoSPARC (162)] to compare the motion of the ECD relative to the TMD; the authors observed significant “rocking and twisting” motion at the ECD, which suggests larger conformational variances at the ECD of GLP-1R when pep-19 is bound versus GLP-1 (157).

Besides amino acid sequence specificity that contributes to the dual and triple agonists’ polypharmacology, the presence of the lipidic acylation moiety also appears to play a salient role (Fig. 3). Acylation on the peptide enhances binding to plasma albumin and extends the peptide half-life in vivo, important for the therapeutic efficacy of these agents; however, the acyl moiety may also impact receptor pharmacology. Tirzepatide exhibits full agonism at the GIPR and G-protein-biased agonism at the GLP-1R, leading to reduced β-arrestin recruitment and receptor internalization (163). However, this unique pharmacology at the GLP-1R can be partially reversed when interrogated with a nonacylated/lipidated version of the peptide (151). For peptide 20, when the lipidic moiety attached to residue K20 is removed, the ligand has a marked reduction (>100-fold) in receptor-mediated cAMP accumulation at the GIPR and the GCGR, while remaining relatively unaffected at activating the GLP-1R (158). At present, the influence of fatty acid modification on the intrinsic pharmacological profile of other advanced molecules, including mazdutide, pemvidutide, and survodutide that contain such moieties, is unreported. Ideally, structural studies and receptor activation assays would be performed with both the ligand and its corresponding unacylated parent peptide.

Taken together, the structural and pharmacological observations coupled with computational analyses via 3DVA and MD simulations on the dual and triple agonist-bound receptor structures indicate that receptor polypharmacology and conformational variance are correlated. The native ligands tend to stabilize their respective receptors at reduced conformational dynamics leading to balanced agonism, whereas dual and triple agonists together with their lipid modifications tend to put the GLP-1R, GIPR, or GCGR in higher conformational dynamics, eliciting unique pharmacological characteristics, including biased or partial agonism.

## CONCLUSIONS

During the past three decades, the identification and characterization of the class B1 subfamily of GPCRs and their respective ligands have improved our understanding of many of the body’s homeostatic mechanisms regulating physiology. There are now several therapeutics that have been discovered that mimic the pharmacological actions of certain native ligands to treat pathophysiological conditions, including drugs for osteoporosis, growth disorders, CNS conditions, such as migraine, and T2D and obesity. For the latter two indications, medicines targeting the GLP-1R have delivered therapeutic benefits such as improved glycemic control and reduced body weight, with generally lower risk of hypoglycemia than has historically been observed with other antidiabetic treatments. Most recently, new multifunctional ligands that activate the GLP-1R and either the GIPR or the GCGR (or both) have emerged, with the GIP and GLP-1 receptor agonist tirzepatide being the first approved medicine of the class. Looking ahead, the next multifunctional agonist seeking regulatory authorization may be an agent containing GCGR activity, a pharmacology predicted to enhance energy expenditure.

Determining the molecular basis for multireceptor agonism of the GLP-1, GIP, and glucagon receptors has been substantially aided by structural biology studies using cryo-EM. Although this work revealed the sufficient homologies of the native ligands for cross-receptor interaction and similarities of the receptor binding pockets of these GPCRs, there remain important questions about the optimal pharmacological characteristics of dual and triple agonists. For example, structural studies of efinopegdutide, mazdutide, pemvidutide, and survodutide in active state receptor complexes are needed, as such data should provide key insights when overlaying with the high-resolution structures of receptor-bound OXM and pep-15. These findings would then be integrated with data from in vitro receptor activation studies assessing potency of the ligands for the GLP-1R and GCGR, and clinical efficacy results from trials investigating these molecules, altogether helping to determine the optimal pharmacological profile for this dual receptor mechanism. This type of iterative approach, along with understanding the influence of additional features such as fatty acid modification, offers potential to maximize the multifaceted benefit of collectively targeting the GLP-1, GIP, and glucagon receptors. Similarly, other class B1 GPCRs, such as the amylin and CRH2 receptors, may provide even further opportunities for developing complementary treatments for metabolic disease, thereby offering the possibility of further expanding the therapeutic potential of the class B1 3GPCRs.

## GRANTS

This work was supported by Eli Lilly and Company (Lilly) Grant 100004312 (to A.T.).

## DISCLOSURES

P.S., J.D.H., and K.W.S. are employees of Eli Lilly and Company and may own company stock. A.T. and B.J. have received Lilly Research Award Program (LRAP) support from Eli Lilly and Company. None of the other authors has any conflicts of interest, financial or otherwise, to disclose.

## AUTHOR CONTRIBUTIONS

P.S. and K.W.S. conceived and designed research; P.S., J.D.H., T.S., J.L., and A.T. prepared figures; P.S., J.D.H., T.S., J.L., A.T., B.J., and K.W.S. drafted manuscript; P.S., J.D.H., T.S., J.L., A.T., B.J., and K.W.S. edited and revised manuscript; P.S., J.D.H., and K.W.S. approved final version of manuscript.
